# Bridging With Low‐Molecular‐Weight Heparin Versus Antiplatelet Therapy in Patients Undergoing Noncardiac Surgery After Percutaneous Coronary Intervention: A Comprehensive Review

**DOI:** 10.1002/clc.70008

**Published:** 2024-09-11

**Authors:** Syed Javaid Iqbal, Zulfiqar Qutrio Baloch, Jahanzeb Malik, Nikeeta Bhimani, Amin Mehmoodi, Vishal Gupta

**Affiliations:** ^1^ Department of Cardiovascular Research Cardiovascular Analytics Group Islamabad Pakistan; ^2^ Department of Interventional Cardiology Ascension Borgess Hospital Michigan USA; ^3^ Department of Medicine Ibn e Seena Hospital Kabul Afghanistan

**Keywords:** anticoagulation, bridging, low‐molecular‐weight‐heparin

## Abstract

**Background:**

This review article discussed the use of bridging therapy with low‐molecular‐weight heparin (LMWH) in patients who undergo noncardiac surgery (NCS) after percutaneous coronary intervention (PCI).

**Hypotheses:**

Patients who undergo PCI are at an increased risk of thrombotic events due to their underlying cardiovascular disease. However, many of these patients may require NCS at some point in their lives, which poses a significant challenge for clinicians as they balance the risk of thrombotic events against the risk of bleeding associated with antithrombotic therapy.

**Results:**

This review evaluates the current evidence on the use of bridging therapy with LMWH in patients undergoing NCS after PCI, focusing on outcomes related to the efficacy and safety of antithrombotic therapy. The article also discusses the limitations of the current evidence and highlights areas where further research is needed to optimize the management of antithrombotic therapy in this patient population.

**Conclusion:**

The goal of this review was to provide clinicians with a comprehensive summary of the available evidence to guide clinical decision‐making and improve patient outcomes.

## Graphical abstract

Pros and cons of bridging in noncardiac surgery patients.

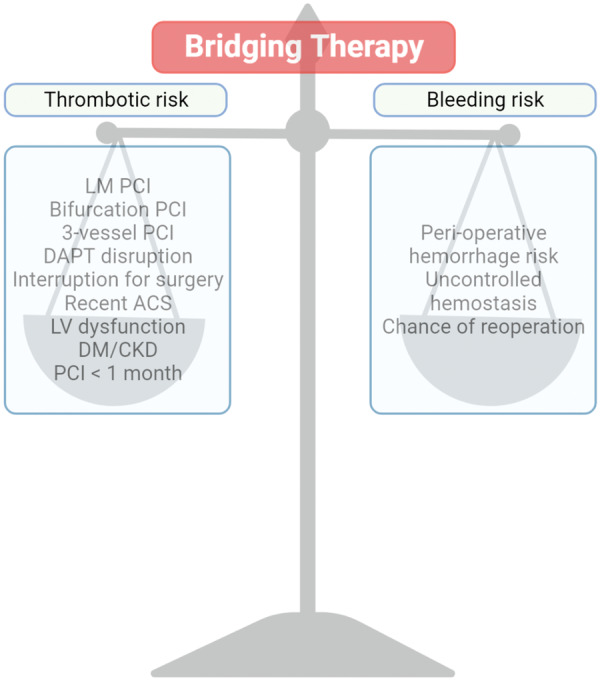

## 1 Introduction

Patients who undergo percutaneous coronary intervention (PCI) are at an increased risk of thrombotic events due to their underlying cardiovascular disease [1]. In addition, many of these patients may require noncardiac surgery (NCS) at some point in their lives, which poses a significant challenge for clinicians as they balance the risk of thrombotic events against the risk of bleeding associated with antithrombotic therapy [2]. Bridging therapy with low‐molecular‐weight heparin (LMWH) has been used as a strategy to reduce the risk of thrombotic events in patients undergoing NCS after PCI [3]. However, the optimal approach to perioperative management of antithrombotic therapy in this patient population remains controversial. Some studies have suggested that bridging therapy with LMWH may be associated with a lower risk of thrombotic events, while others have reported a higher risk of bleeding complications [3, 4, 5, 6, 7].

The pathophysiology of thrombotic events in patients with cardiovascular disease is complex and multifactorial [8]. PCI, which involves the placement of stents in coronary arteries, is a common intervention for the management of coronary artery disease [9]. However, stent placement can lead to endothelial injury, platelet activation, and activation of the coagulation cascade, all of which contribute to the risk of thrombotic events [10]. The use of antiplatelet therapy, such as aspirin and P2Y12 inhibitors, is standard practice in patients undergoing PCI to reduce the risk of thrombotic events [11]. However, the optimal management of antithrombotic therapy in the perioperative period of NCS remains uncertain. Interruption of antiplatelet therapy increases the risk of thrombotic events, while continuation of therapy increases the risk of bleeding complications [12]. Bridging therapy with LMWH has been proposed as a strategy to reduce the risk of thrombotic events during the perioperative period while minimizing the risk of bleeding complications [13].

**Figure 1:**
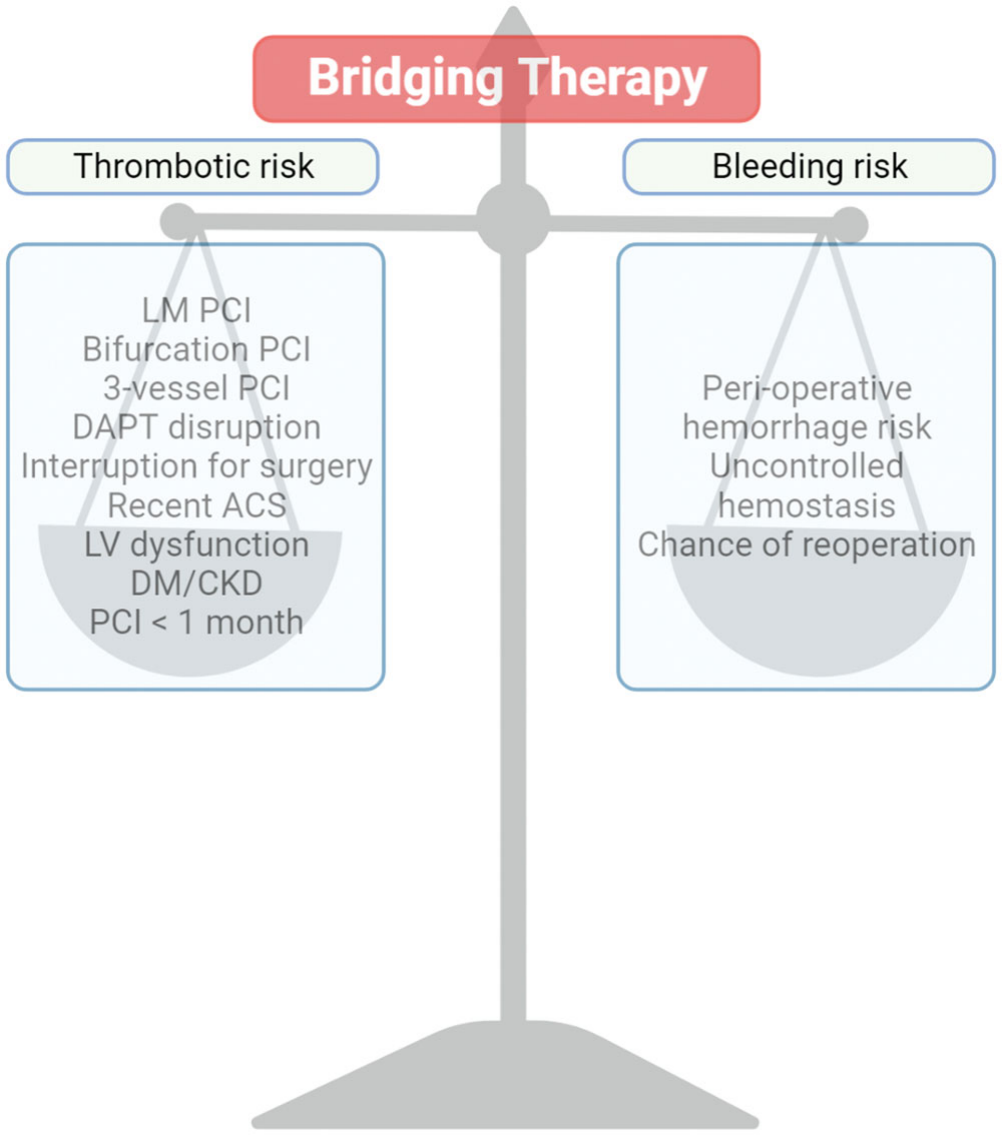
Risk‐benefit between bleeding and thrombotic risk in patients undergoing noncardiac surgery with post‐PCI.

This review will evaluate the current evidence on the use of bridging therapy with LMWH in patients undergoing NCS after PCI, focusing on outcomes related to the efficacy and safety of anticoagulant and antithrombotic therapy. The review will also discuss the limitations of the current evidence and highlight areas where further research is needed to optimize the management of antithrombotic therapy in this patient population. Ultimately, the goal of this review is to provide clinicians with a comprehensive summary of the available evidence to guide clinical decision‐making and improve patient outcomes (Figure 1).

## 2 Methods

A comprehensive literature search was conducted using electronic databases such as PubMed, Embase, and Cochrane Library. The search terms used were “bridging,” “low‐molecular‐weight heparin,” “noncardiac surgery,” “percutaneous coronary intervention,” “PCI,” and “review.” The search was restricted to studies published in English from inception to the present time. The studies were assessed for eligibility by two independent reviewers, and any discrepancies were resolved by consensus. The inclusion criteria were studies that investigated the use of LMWH as bridging therapy in patients undergoing NCS after PCI. Studies that did not meet the inclusion criteria or were of low quality were excluded from the review. Data were extracted from the included studies, and the findings were synthesized in a narrative fashion.

## 3 Main Text

### 3.1 The Rationale for Bridging After PCI

The rationale for bridging after PCI is to prevent the development of thrombotic events in the perioperative period [14]. Patients who have undergone PCI are at an increased risk of developing thrombotic events due to the presence of coronary artery stents and the disruption of the vessel wall [15]. The discontinuation of antiplatelet therapy in the perioperative period further increases the risk of thrombotic events [16]. Bridging therapy with LMWH is believed to prevent thrombotic events by inhibiting the activity of thrombin and factor Xa, which are key enzymes involved in the coagulation cascade [17]. LMWH has a higher affinity for factor Xa compared to thrombin, which results in the inhibition of thrombin generation and the prevention of clot formation [18]. Additionally, LMWH enhances the activity of antithrombin III, which is a natural anticoagulant that inhibits thrombin and factor Xa [19]. LMWH is preferred over unfractionated heparin (UFH) for bridging therapy due to its longer half‐life, lower risk of heparin‐induced thrombocytopenia, and more predictable anticoagulant effect [20]. However, bridging therapy with LMWH is associated with an increased risk of bleeding, which needs to be carefully balanced against the risk of thrombotic events in each patient [21]. Peri‐procedural ischemic complications and their risk factors are tabulated in Table 1.

**Table 1:** Factors associated with increased peri‐procedure ischemic complications.

Clinical	Procedural
Multivessel disease	Multivessel stenting
Chronic kidney disease	> 3 stents in situ
Diabetes mellitus	> 3 lesions treated
Left ventricular dysfunction	Bifurcation stenting
Revised cardiac risk index > 3	Stent length of > 60 mm
Acute coronary syndrome	Left main stem stenting
Prior stroke	High SYNTAX

### 3.2 Pathophysiology of Thrombotic Events

Thrombotic events occur due to the pathophysiological changes that occur within the cardiovascular system, leading to the formation of clots within the blood vessels [22]. The pathophysiology of thrombotic events in patients with cardiovascular disease involves several factors that contribute to the formation of blood clots, including endothelial dysfunction, platelet activation, and the coagulation cascade [23, 24, 25]. Endothelial dysfunction is a critical factor in the pathophysiology of thrombotic events [23]. The endothelium is a single layer of cells that line the inner surface of blood vessels. It plays a crucial role in maintaining vascular homeostasis by regulating the release of vasoactive substances and preventing platelet activation and adhesion [26]. In patients with cardiovascular disease, the endothelium becomes damaged due to the presence of risk factors such as hypertension, hyperlipidemia, and diabetes mellitus [27]. This damage leads to the activation of the clotting cascade, platelet adhesion, and thrombus formation [26, 27]. Platelet activation is another critical factor in the pathophysiology of thrombotic events [24]. Platelets are small, anucleate cells that play a crucial role in hemostasis. In patients with cardiovascular disease, platelets become activated due to the presence of atherosclerotic plaques or the release of inflammatory cytokines [28]. Activated platelets aggregate and release granules containing thromboxane A2 and serotonin, which promote vasoconstriction and platelet activation, leading to the formation of a thrombus [29]. The coagulation cascade is the final critical factor in the pathophysiology of thrombotic events [25]. The coagulation cascade is a complex series of enzymatic reactions that result in the formation of a fibrin clot [30]. In patients with cardiovascular disease, the coagulation cascade becomes activated due to the presence of damaged endothelium and activated platelets [31]. The activation of the coagulation cascade leads to the conversion of prothrombin to thrombin, which cleaves fibrinogen to fibrin, resulting in the formation of a thrombus [31].

### 3.3 Literature Review on Bridging Therapy

Bridging antiplatelet therapy with LMWH in patients with prior stent implantation who undergo noncardiac surgical procedures is a common practice. The rationale behind this practice is to prevent stent thrombosis, which can lead to myocardial infarction, stroke, or death. Patients who have received a stent for the treatment of coronary artery disease are typically prescribed dual antiplatelet therapy (DAPT), consisting of aspirin and a P2Y12 receptor inhibitor such as clopidogrel, prasugrel, or ticagrelor [32]. These medications help prevent stent thrombosis by inhibiting platelet aggregation. However, when these patients require NCS, there is a risk of bleeding associated with continued DAPT use [33]. This has led to the practice of temporarily interrupting DAPT before surgery, typically for 5–7 days. However, this interruption increases the risk of stent thrombosis [34]. To mitigate this risk, some clinicians choose to bridge the patient with LMWH during the perioperative period. LMWH is effective in preventing stent thrombosis, and its shorter half‐life compared to antiplatelets makes it more easily reversible in the event of bleeding [35]. However, one study found that discontinuation of antiplatelet therapy, and LMWH bridging was associated with a 10−12 fold increase in the risk of MACE compared to continuation of antiplatelets with a twofold increase in BARC bleeding [3]. This retrospective analysis examined 515 patients with coronary stents undergoing NCS, half of whom received LMWH bridging. At 30 days, those who received LMWH had a higher incidence of major adverse cardiac or cerebrovascular events (7.2% vs. 1.1%) and myocardial infarction (4.8% vs 0%). Additionally, they faced a greater risk of significant bleeding (21.9% vs. 11.7%). These findings were consistent across various statistical adjustments and propensity matching. In conclusion, LMWH bridging in these patients may lead to worse ischemic outcomes and increased bleeding risks [3]. The impact of discontinuation of antiplatelets in stented patients undergoing surgery has conflicting results as several considerations are confounding the interpretation of the available literature, including varied cardiac risk, type of revascularization, stent type, PCI time, individuals risk of bleeding versus thrombosis, and impact of different modalities of antiplatelet and anticoagulation [33]. The recent guidelines do not provide clear guidance on the management of post‐PCI antiplatelet therapy about different types of interventions, their risk of stent thrombosis, and bleeding [36].

The guidelines discourage the use of LMWH for bridging patients on antiplatelet therapy for NCS, but both documents do not provide a formal class III recommendation and acknowledge the paucity of literature in this subset of patients [36, 37]. This increased the ischemic events given the withdrawal of protection from antiplatelet therapy. Furthermore, bleeding itself has been associated with both discontinuations of antiplatelet therapy and ischemic events [38]. LMWH does not stimulate platelet aggregation like UFH, but it also does not have a significant platelet inhibitory effect in preventing stent thrombosis or myocardial infarction [19]. The impact of LMWH on bleeding is observed to be biologically plausible and raises a point of concern in the diffuse practice of switching from antiplatelet to LWMH. if it is necessary to bridge a patient, an antiplatelet should be preferred as compared to anticoagulants. Other major studies involving the use of antiplatelets in NCS or bridging with LMWH are shown in Table 2 [3, 39, 40, 41, 42, 43, 44, 45].

**Table 2:** Current studies on bridging with LMWH versus use of antiplatelet in noncardiac surgery.

References	Country	Participants	Type of intervention	Outcome	Comment
*Studies involving antiplatelet use in noncardiac surgery*					
Antolovic et al. [39]	Germany	52	Use of aspirin in abdominal surgery	One patient (3.8%) in the ASA continuation group required reoperation due to postoperative hemorrhage. In neither study group, further bleeding complications occurred. No clinically apparent thromboembolic events were reported in the ASA continuation and the ASA discontinuation group. Furthermore, there were no significant differences between both study groups in the secondary endpoints.	Perioperative intake of ASA does not seem to influence the incidence of severe bleeding in non–high‐risk patients undergoing elective general or abdominal surgery. Further, adequately powered trials are required to confirm the findings of this study.
Chu et al. [40]	USA	48	Use of clopidogrel in elective general surgery	No perioperative mortalities, bleeding events requiring blood transfusion, or reoperations occurred. One readmission for intra‐abdominal hematoma requiring percutaneous drainage occurred in each group (group A: 4.8% vs. group B: 4.5%; *p* = 1.0). No myocardial infarctions or cerebrovascular accidents were observed or reported.	The outcomes suggest that, patients undergoing commonly performed elective general surgical procedures can be safely maintained on clopidogrel without increased perioperative bleeding risk.
Mantz et al. [41]	France	291	Use of aspirin in urologic, orthopedic, and general surgery	No significant difference was observed neither in the primary outcome score [mean values (SD) = 0.67 (2.05) in the aspirin group versus 0.65 (2.04) in the placebo group, *p* = 0.94] nor at day 30 in the number of major complications between groups.	In these at‐risk patients undergoing elective noncardiac surgery, we did not find any difference in terms of occurrence of major thrombotic or bleeding events between preoperative maintenance or interruption of aspirin.
Nielsen et al. [42]	Denmark	55	Use of aspirin TURP	There was no significant difference in the median operative blood loss between the groups (*p* = 0.528), but postoperatively the blood loss in the ASA group (median 284; quartiles 196–660 mL) was significantly higher than in the placebo group (median 144; quartiles 75–379 mL), (*p* = 0.011).	Long‐term low‐dose ASA therapy is associated with a significant increase in the postoperative blood loss after TURP.
*Studies involving bridging with LMWH in noncardiac surgery*					
Hart et al. [43]	Netherland	238	Bridging with LMWH versus UFH in noncardiac surgery	The incidence of major bleeding was 16 (19%) events in the UFH group versus 29 (19%) events in the LMWH group (*p* = 0.97). Incidences of thromboembolism were 2 (2.4%) versus 1 (0.6%). The incidence of death was 1 (1.2%) patient in the UFH group versus 3 (1.9%) patients in the LMWH group. More than 50% of all bleeding complications were categorized as a major bleeding.	Bridging anticoagulation in patients with aortic and mitral mechanical valves is associated with considerable risk, but no difference was apparent between UFH and LMWH strategy.
Spyropoulos et al. [44]	USA	245	Bridging with LMWH versus UFH in noncardiac surgery	Major adverse event rates (5.5% vs. 10.3%, *p* = 0.23) and major bleeds (4.2% vs. 8.8%, *p* = 0.17) were similar in the LMWH and UFH groups, respectively; 1 arterial thromboembolic event occurred in each group. More LMWH‐bridged patients were treated as outpatients or discharged from the hospital in < 24 h (68.6% vs. 6.8%, *p* < 0.0001).	For patients with mechanical prosthetic heart valves on long‐term OAT, mostly outpatient‐based LMWH bridging therapy appears to be feasible for selected procedures, is as safe as UFH, and is associated with a low arterial thromboembolic rate.
Capodanno et al. [3]	Italy	515	Bridging of LMWH in post‐PCI patients undergoing noncardiac surgery	At 30 days, MACCE occurred more frequently in patients who received LMWH (7.2% vs. 1.1%, *p* = 0.001), driven by a higher rate of myocardial infarction (4.8% vs. 0%, *p* < 0.001). This finding was consistent across several instances of statistical adjustment and after the propensity matching of 179 pairs. Patients bridged with LMWH also experienced a significantly higher risk of BARC bleedings ≥ 2 (21.9% vs. 11.7%, *p* = 0.002) compared to those who were not.	LMWH bridging in patients with coronary stents undergoing surgery is a common and possibly harmful practice, resulting in worse ischemic outcomes at 30 days, and a significant risk of bleeding.
Angiolillo et al. [45]	United states	11	To evaluate the use of cangrelor, an intravenous, reversible P2Y12 platelet inhibitor for bridging thienopyridine‐treated patients to coronary artery bypass grafting	In the study, a dose of 0.75 µg/kg/min of cangrelor was established during the open‐label phase. In the randomized phase, significantly more patients treated with cangrelor maintained low platelet reactivity levels throughout the treatment compared to those on placebo (primary endpoint, PRU < 240: 98.8% vs. 19%; RR: 5.2, 95% CI: 3.3–8.1, *p* < 0.001). Excessive bleeding related to CABG surgery occurred in 11.8% of the cangrelor group and 10.4% of the placebo group (RR = 1.1, 95% CI: 0.5–2.5, *p* = 0.763), showing no significant difference. While major bleeding before CABG was not significantly different between the groups, minor bleeding was slightly higher with cangrelor.	Among patients who must wait for cardiac surgery after thienopyridine discontinuation, the use of cangrelor compared with placebo resulted in a higher rate of maintenance of platelet inhibition.

### 3.4 Expert Opinion

#### 3.4.1 Clinical Implications

The use of LMWH in this population is associated with an increased risk of bleeding, which may outweigh the benefits of thromboprophylaxis [3, 43, 44]. Therefore clinicians need to consider the benefits and risks of bridging therapy and make an informed decision based on the individual patient's situation. The risk of bleeding is higher in patients who are elderly, have a history of bleeding, have renal insufficiency, or are on concomitant antithrombotic therapy. Therefore, clinicians should consider this risk and assess the patient's bleeding risk before deciding to use bridging therapy.

It may benefit high‐risk patients undergoing NCS after PCI, such as those with a recent myocardial infarction or those with a high thrombotic risk. In these patients, the benefit of thromboprophylaxis may outweigh the bleeding risk associated with bridging therapy.

The decision to use bridging therapy with LMWH should be individualized based on the patient's thrombotic and bleeding risk. Clinicians should weigh the benefits and risks of bridging therapy and make an informed decision based on the individual patient's situation. This decision should be based on factors such as the type of surgery, the timing of surgery, the patient's bleeding risk, and the patient's thrombotic risk.

The timing of bridging therapy with LMWH is important. It should be initiated early enough to provide adequate thromboprophylaxis, but late enough to minimize the bleeding risk associated with the procedure. The timing of therapy may depend on the type of surgery, the patient's bleeding risk, and the patient's thrombotic risk.

Communication between healthcare providers is essential to ensure that the patient's antithrombotic therapy is managed appropriately during the perioperative period. Clinicians should ensure that the patient's primary care provider, cardiologist, and surgeon are aware of the patient's antithrombotic therapy and that they are involved in the decision‐making process regarding bridging therapy. This can help to ensure that the patient's antithrombotic therapy is managed appropriately during the perioperative period and that the risk of bleeding is minimized.

### 3.5 Future Directions

Although bridging therapy with LMWH is a common practice in patients undergoing NCS after PCI, there are still several unanswered questions regarding its use. Therefore, future research should focus on addressing these questions and improving the management of these patients. The following are the detailed future directions for research on bridging with LMWH in patients undergoing NCS after PCI.

(1) Determining the optimal timing of bridging therapy: The optimal timing of bridging therapy with LMWH is not well‐defined, and there is a need for further research in this area. Future studies should evaluate the timing concerning the relation to the type of surgery, the patient's thrombotic risk, and the patient's bleeding risk. This information can help to guide clinicians in the management of these patients.
(2) Assessing the bleeding risk associated with bridging therapy: Although the use of bridging therapy with LMWH is associated with an increased risk of bleeding, the exact risk is not well‐defined. Future studies should evaluate the bleeding risk associated with bridging therapy in patients undergoing NCS after PCI. This information can help to inform the decision‐making process regarding the use of bridging therapy in these patients.
(3) Evaluating the efficacy of alternative antithrombotic therapies: There is a need for further research on alternative antithrombotic therapies that may be used instead of bridging therapy with LMWH in patients undergoing NCS after PCI. For example, studies could evaluate the efficacy of antiplatelet therapy alone in these patients. This information can help to guide the management of these patients and improve outcomes.
(4) Assessing the impact of bridging therapy on long‐term outcomes: Although bridging therapy with LMWH may prevent thrombotic events in the perioperative period, its impact on long‐term outcomes is unclear. Future studies should evaluate the impact of bridging therapy on long‐term outcomes, such as mortality and major adverse cardiovascular events, in patients undergoing NCS after PCI.
(5) Developing guidelines for the management of these patients: There is a need for the development of guidelines for the management of patients undergoing NCS after PCI. These guidelines should take into consideration the patient's thrombotic and bleeding risk, the type of surgery, and the timing of surgery. Developing guidelines can help to standardize the management of these patients and improve outcomes.

## 4 Conclusion

In conclusion, the use of bridging therapy with LMWH in patients with prior stent implantation undergoing NCS is a complex issue with conflicting results in the literature. The rationale behind bridging is to prevent stent thrombosis, but it comes with an increased risk of bleeding. LMWH is preferred over UFH due to its longer half‐life and more predictable anticoagulant effect. However, its use in this population is associated with an increased risk of bleeding, which may outweigh the benefits of thromboprophylaxis. The decision to use bridging therapy with LMWH should be individualized based on the patient's thrombotic and bleeding risk.

## Conflicts of Interest

The authors declare no conflicts of interest.

## Data Availability

Data sharing not applicable to this article as no data sets were generated or analyzed during the current study.
